# Assessment of weight-related factors of adolescents by private practitioners

**DOI:** 10.1186/1471-2296-14-141

**Published:** 2013-09-26

**Authors:** Rong Huang, Sai Yin Ho, Wing Sze Lo, Tai Hing Lam

**Affiliations:** 1 School of Public Health, The University of Hong Kong, 21 Sassoon Road, Pokfulam, Hong Kong

**Keywords:** Adolescent, Assessment, Body weight, Private practitioners

## Abstract

**Background:**

Few studies have examined how common physicians assess various weight-related variables and patient characteristics that predict such assessments based on adolescents’ reports. We aimed to examine how common adolescents received weight-related physical measurements and lifestyle enquiries (dietary habits and physical activity) from private practitioners and to identify factors associated with these assessments.

**Methods:**

In the Hong Kong Student Obesity Surveillance (HKSOS) project, 33692 students (44.9% boys; mean age 14.8, SD 1.9 years, age range 11–18) from 42 randomly selected schools completed an anonymous questionnaire. The students were asked “In the past 12 months, has any private practitioners (or their nurses) measured or asked about these items?” Response options included height, weight, waist circumference (WC), blood pressure (BP), BMI, diet, and physical activity. Weight status was based on self-reported weight and height. Logistic regression was used to identify student characteristics associated with each assessment. Analyses were conducted using STATA 10.0.

**Results:**

Among 13283 students who had doctor consultations in the past 12 months, 37.9% received physical measurements or lifestyle enquiries, with weight (20.8%), height (16.8%) and blood pressure (11.5%) being the most common, followed by diet (8.1%), BMI (6.3%), WC and physical activity (both 4.6%). In general, adolescents who were female, older, underweight or overweight/obese, had parents with higher education level, and had actively asked private practitioners for advice about weight were more likely to receive assessments of weight-related factors.

**Conclusions:**

Weight-related factors in adolescents were infrequently assessed by private practitioners in Hong Kong. Generally, unhealthy weight, higher parental education and advice-seeking by adolescents predicted these assessments.

## Background

Childhood obesity is a public health concern worldwide due to serious health consequences in adulthood such as cardiovascular diseases, type 2 diabetes, and certain cancer, which calls for better surveillance and prevention [1]. It is notable that recent evidence even linked childhood obesity with endothelial changes and vascular damage [2]. In addition, excess weight and underweight in adolescents are associated with poor psychological health due to weight stigma, discrimination, and body dissatisfaction [3-7], although such associations vary by sex, race-ethnicity, and culture [8,9]. Given the severe hazards of unhealthy weight, it is important for adolescents to have a healthy weight and be informed of their own weight status.

Unlike adults who have definite body mass index (BMI) cutoffs for weight status to follow, growing children necessitate the use of age- and sex-specific BMI cutoffs [10] or weight-for-height cutoffs [11,12] to define underweight and overweight/obesity. A simpler indicator of weight status using the ratio of waist circumference to height was only recently proposed [13]. Many adolescents find it difficult to evaluate their own weight status as reflected by widespread weight misperception, which often leads to body dissatisfaction and poor psychosocial health [14].

Primary care physicians are usually the first contact of paediatric patients. About 58% of Canadian adolescents have sought medical consultation in the previous 6 months [15], so doctors have the opportunity to assess their weight status, offer advice and clarify weight misperception. Indeed, guidelines issued by the American Medical Association, the Health Resources and Service Administration, and the Centers for Disease Control and Prevention, have recommended primary care providers to universally calculate and plot BMI to record weight status and assess obesity-related medical risk, discuss dietary and physical activity habits with children and adolescents [16,17]. Physicians’ or health professionals’ acknowledgment of overweight status have shown to be associated with attempts to lose weight in overweight adolescents and adults [18,19]. However, the assessment of weight status and counselling on lifestyle by primary care physicians are often inadequate, mainly due to heavy clinical load, lack of patient motivation, and lack of parent involvement [20-23]. Emerging studies have shown that weight management practice in office-based setting varied by patients’ sex, age, weight status, and physicians’ personal characteristics and career type [20,24-26]. Little is known about the association between socio-demographic characteristics of adolescent patients and each weight-related assessment.

In Hong Kong, medical services are mainly provided by public healthcare services under the Hospital Authority (public hospitals, general/specialist out-patient clinics, Chinese medicine services), private hospitals, and private clinics [27]. Private clinics are many and easily accessible in Hong Kong. More than half of all medical consultations are conducted by their private practitioners of Western medicine (referred to as private practitioners hereafter) [28]. Private practitioners are the first point of contact for most Hong Kong adolescent patients. We investigated how common Hong Kong adolescents received assessments of each weight-related factor by private practitioners, including height, weight, BMI, waist circumference, weight status, blood pressure, exercise and diet, and identified adolescent characteristics associated with these assessments.

## Methods

### Study design

The present study was part of a school-based study in 2006–7, the Hong Kong Student Obesity Surveillance (HKSOS) project. Stratified sampling was applied to select 42 schools by district, source of funding, language of instruction (Chinese/English), religious background (Christian/Others/None) and single sex/co-education to represent all main-stream non-international secondary schools in Hong Kong [14]. All secondary 1 (US grade 7) to secondary 7 students (mean age 14.8, SD 1.9 years, age range 11–18) in the selected schools were invited to participate.

### Ethical consideration

The schools acted *in loco parentis* for the students. Parents are informed through an invitation letter and agreement to participate in the survey was assumed unless they returned an included reply slip. Student participation remained voluntary even with parental consent. Ethical approval was granted by the Institutional Review Board of the University of Hong Kong/Hospital Authority Hong Kong West Cluster.

### Subjects

A total of 33692 students aged 11–18 years completed an anonymous questionnaire in classrooms. Three questions were used to collect information on doctor consultation in the past 12 months and 13283 (39.4%) students who consistently reported having doctor consultations in the 3 questions were included.

### Measures

Students were asked “Did you receive any of the following measurements or enquiries from private practitioners or their nurses in the past 12 months”. Response options included: did not see a private practitioner, height, weight, BMI, waist circumference, blood pressure, physical activity and diet. Similarly, students reported whether any private practitioners had commented on their weight status and whether they had actively asked any private practitioners for advice on body weight in the past 12 months.

Self-reported height and weight were used to define weight status (underweight, normal weight, overweight, and obesity) using the International Obesity Taskforce standard (IOTF) [10,29]. The sex-and age-specific BMI cutoff values from IOTF standards of defining overweight and obesity are set to align with adult BMI values of 25 Kg/m^2^ and 30 Kg/m^2^, respectively. These standards were created using anthropometric data from 6 nationally representative samples from Brazil, Great Britain, Hong Kong, the Netherlands, Singapore, and the United States. Underweight status was defined based on BMI cut-off values established in 2007. Based on the same dataset, we have previously reported good correlation between self-reported and measured height and weight in both boys and girls [30]. Since among 13283 students, only 132 (0.99%) students were identified as obese based on the BMI cutoff values of classifying obesity according to IOTF standard, overweight and obesity were grouped together in statistical analysis. Sex, age and parental education levels (primary or below/secondary/tertiary or above) were also reported.

### Data analysis

Our outcomes are whether adolescents received assessment of each weight-related factor, including height, weight, BMI, waist circumference, blood pressure, physical activity, and diet, and the exposures are adolescent sex, age, weight status, highest parental education, and whether adolescents actively asked private practitioners for advice on weight. Chi-square tests were used to test the bivariate association between each assessment and each exposure. Factors significantly associated with the assessment in Chi-square test (p < 0.05) were included in logistic regression, which was used to compute adjusted OR with 95% confidence interval (CI) of each assessment for each exposure variable.

Only 64% of the original sample (n = 13283) had complete data on all regression variables, and 36% of the sample had missing values in one or more variables in the regression: weight status (27.1%), highest parental education (12.8%), and other variables (2.6%) We compared the socio-demographic characteristics of subjects with and without missing data. Those without missing data were more likely to be girls (58.0% vs 53.3% in those with missing data), older (14.5 vs 14.4 years), underweight (8.5% vs 6.9%) and had parents with teritary education (17.1% vs 11.7%), representing small to medium differences (Cohen’s effect size ranged from 0.02 to 0.16). Additionally, because reduced sample size may result in inefficient estimates and inflated standard error, we used multiple imputation, which was proposed by Rubin in 1987 [31], to impute the missing data. Each missing value is imputed 10 times to account for the uncertainty underlying the missing values using “aregImpute” function in the “Hmisc” package in R based on additive regression, bootstrapping, and predictive mean matching [32]. The imputation model included variables of the logistic regression models and auxiliary variables in the database. We conducted separate logistic regression analysis for each imputed dataset, and then combined the estimates based on Rubin’s rule [31]. Sensitivity analysis showed that these results were comparable to those based on complete-case analysis. All statistical analyses were conducted using STATA 10.0 (Stata Corporation, College Station, TX) and R.

## Results

Table 1 shows that of 13,283 adolescents who had doctor consultations in the past 12 months, 8.5% were underweight and 9.8% were overweight. Only 1 in 10 students had asked a private practitioner for advice on body weight. About one-third received private practitioners’ comments on weight status. Weight was most commonly assessed (20.8%), followed by height (16.8%), blood pressure (BP) (11.5%), diet (8.1), BMI (6.3%), waist circumference and physical activity (both 4.6%).

**Table 1:** Characteristics of subjects who had doctor consultation in the past 12 months (n = 13283)

**Characteristics**	**n (%)**
Sex	
Boys	5529 (42.0)
Girls	7625 (58.0)
Age group	
<=14	6790 (51.1)
>14	6493 (48.9)
Weight status	
Underweight	821 (8.5)
Normal weight	7909 (81.7)
Overweight	950 (9.8)
Highest parental education	
Primary or below	1512 (13.0)
Secondary	8149 (69.2)
Tertiary or above	1993 (17.1)
Actively asked private practitioners for advice on weight	
Yes	1276 (9.8)
No	11788 (90.2)
Private practitioners commented on adolescents’ weight status	
Yes	3784 (28.7)
No	9413 (71.3)

*Abbreviations*: BMI, body mass index; WC, waist circumference; BP, blood pressure.

Table 2 shows that weight-related items were generally more commonly assessed in adolescents who had actively asked a private practitioner for advice on body weight, or had parents with tertiary or above education. Additionally, girls were more likely than boys to receive assessments of height, weight, blood pressure, physical activity, diet and weight status, while girls were less likely to receive assessments of BMI and waist circumference. Older adolescents were more likely to have their BP assessed but less likely to have their height and diet assessed. Underweight adolescents were more likely to have their height and weight assessed, but overweight adolescents were more likely to have their BMI and WC assessed.

**Table 2:** Prevalence of each assessment by adolescent characteristics

**Characteristics**	**Prevalence of assessment by private practitioners (%)**							**Private practitioners commented on weight status (3812)**
	**Height (2230)**	**Weight (2757)**	**BMI (842)**	**WC (610)**	**BP (1527)**	**Physical activity (612)**	**Diet (1069)**	
Sex								
Boys	14.3	18.0	7.2	5.8	8.9	4.1	5.6	34.7
Girls	18.7	22.7	5.7	3.7	13.4	4.9	9.9	24.1
P value	< 0.001	< 0.001	0.001	< 0.001	< 0.001	0.033	< 0.001	< 0.001
Age group								
<=14	17.6	21.4	6.4	4.5	9.2	4.6	8.5	30.4
>14	16.0	20.1	6.3	4.7	13.9	4.6	7.6	26.9
P value	0.012	0.082	0.691	0.629	< 0.001	0.879	0.038	< 0.001
Weight status								
Underweight	21.8	24.4	6.8	4.8	14.1	5.9	10.6	33.6
Normal weight	16.8	20.2	4.9	3.5	12.0	4.3	8.4	22.1
Overweight	15.7	22.1	7.0	5.5	11.3	4.5	7.8	31.8
P value	0.001	0.012	0.002	0.005	0.148	0.128	0.073	< 0.001
Highest parental education								
Primary or below	14.5	19.2	5.9	4.1	13.1	4.0	7.7	26.4
Secondary	16.9	20.5	5.6	4.0	11.8	4.5	8.4	26.4
Tertiary or above	20.1	24.5	9.4	6.6	12.2	6.1	9.1	36.9
P value	< 0.001	< 0.001	< 0.001	< 0.001	0.357	0.004	0.341	< 0.001
Actively asked private practitioners for advice on weight								
No	15.1	19.2	5.0	3.7	10.9	4.1	7.4	22.2
Yes	32.7	34.3	16.9	12.4	17.4	9.5	14.7	83.8
P value	< 0.001	< 0.001	< 0.001	< 0.001	< 0.001	< 0.001	< 0.001	< 0.001
Total	16.8	20.8	6.3	4.6	11.5	4.6	8.1	28.7

P values based on Chi-square tests.

*Abbreviations*: BMI, body mass index; WC, waist circumference; BP, blood pressure.

Table 3 shows that all weight-related items were significantly more likely to be assessed in adolescents who had actively asked a private practitioner for advice on body weight. The strongest association was observed for BMI with an adjusted odds ratio (AOR, 95% CI) of 4.14 (3.23, 5.31). Highest parental education was linearly associated with assessments of height (p < 0.001), weight (p = 0.001), BMI (p = 0.015), WC (p = 0.014), as well as physical activity (p = 0.014). Also, underweight was associated with height assessment (p = 0.007) and weight (p = 0.048), while overweight was associated with assessments of weight (p = 0.023), BMI, and WC. Female students were more likely to be assessed for height (p < 0.001), weight (p < 0.001), blood pressure (p < 0.001), physical activity (p = 0.003), and diet (p < 0.001), but less likely for WC (p = 0.018). In addition, older students were more likely to be assessed for BP (p < 0.001), but less likely to be assessed for height (0.007) and diet (0.011).

**Table 3:** Adjusted odds ratio (AOR) of weight-related assessments by private practitioners by adolescent characteristics

	**Adjusted OR (95% CI)**						
	**Height**	**Weight**	**BMI**	**WC**	**BP**	**Physical activity**	**Diet**
Sex							
Boy	1	1		1	1	1	1
Girl	1.36 (1.20, 1.54)**	1.47 (1.31, 1.65)**	0.99 (0.81, 1.21)	0.75 (0.60, 0.95)*	1.65 (1.47, 1.85)**	1.32 (1.10, 1.59)**	1.97 (1.71, 2.27)**
Age group							
<=14	1	NA	NA	NA	1	NA	1
>14	0.85 (0.76, 0.96)**				1.59 (1.42, 1.77)**		0.85 (0.75, 0.96)*
Weight status							
Normal weight	1	1	1	1	NA	NA	NA
Underweight	1.30 (1.07, 1.58)**	1.20 (1.00, 1.44)*	1.36 (0.99, 1.87)	1.39 (0.96, 2.66)			
Overweight	0.94 (0.77, 1.14)	1.23 (1.03, 1.47)*	1.43 (1.06, 1.93)*	1.51 (1.08, 2.10)*			
Highest parental education							
Primary or below	1	1	1	1	NA	1	NA
Secondary	1.19 (0.99, 1.43)	1.10 (0.93, 1.30)	1.14 (0.83, 1.57)	1.30 (0.88, 1.92)		1.10 (0.83, 1.46)	
Tertiary	1.50 (1.20, 1.86)**	1.38 (1.13, 1.68)**	1.53 (1.06, 2.22)*	1.70 (1.09, 2.66)*		1.44 (1.04, 1.98)*	
P for linear trend	< 0.001	0.001	0.015	0.014		0.014	
Actively asked private practitioners for advice on weight							
No	1	1	1	1	1	1	1
Yes	3.23 (2.70, 3.87)**	2.83 (2.37, 3.37)**	4.14 (3.23, 5.31)**	2.75 (2.02, 3.75)**	1.86 (1.59, 2.19)**	2.55 (2.04, 3.20)**	2.45 (2.06, 2.92)**

NA, not applicable; characteristics from chi-square test with P ≤ 0.05 were included in multiple logistic regression.

**P value <0.01; *P value < 0.05. *Abbreviations*: BMI, body mass index; WC, waist circumference; BP, blood pressure.

## Discussion

Around 20% of adolescents had unhealthy weight in our study population. Nearly 30% received weight status comments from private practitioners, but only 6.3% received BMI assessments. In contrast, 60% of primary care physicians reported regularly calculating BMI of children aged 2–17 years in the US [20]. The lower prevalence of BMI assessment in Hong Kong may partly be due to its lower percentage of overweight adolescents [14,33], who generally receive more clinical attention than normal weight adolescents [21,22].

In Hong Kong, the family doctor system is not well developed and doctor shopping is very common [34,35]. Private practitioners were responsible for 70% of outpatient consultations and will be the predominant force of primary care providers if more are switched to family doctors as supposed by recent health care reform [36,37]. The inadequate assessments of weight-related factors suggest that Hong Kong private practitioners should be educated and directed to realize their pivotal role of promoting a healthy weight in adolescents as family doctors [36,38].

Despite the lower assessment rates of weight-related factors, many adolescents received weight comments from private practitioners. This implied some of these weight comments were not based on objective anthropometric measurements, such as BMI. BMI was often under-used despite its usefulness [39]. However, doctors may misclassify patients’ weight status [14], and should be encouraged to assess weight status objectively. The incorrect weight status comments from private practitioners may reflect the insufficient use of objective anthropometric measures in patients due to various barriers, such as time constraints.

We also note that WC was seldom assessed especially in our female adolescents. It is possible that some practitioners feel uncomfortable about carrying out WC measurements and some perceived that patients might feel embarrassed [40]. But one study showed that WC was useful in identifying Chinese adolescents with high cardiovascular risk [41], and the age- and sex- specific waist-to-stature ratio cutoff values have recently been developed for HK adolescents [13], highlighting the need of measuring WC among adolescents.

In general, girls were more likely to receive assessments of weight-related factors. Consistently, girls were more likely to report having discussed body image with their physicians [42]. Similarly, we observed a greater proportion of girls than boys received each assessment except WC and BMI. Doctors probably know that more adolescent girls than boys have weight concerns and therefore more willing to address their concerns [42,43].

Our findings that all the assessments were more likely to occur among those who had actively asked a private practitioner for advice on body weight indicated that students’ weight concern encouraged assessments by doctors and was indirectly supported by findings that lack of patient motivation has been one of the most frequently reported barriers by doctors to participating in weight control of pediatric patients [23]. By actively asking, adolescent patients may help doctors perceive greater weight concerns and greater motivation of changing behaviours, and therefore, doctors are more motivated to perform weight-related assessments [44]. However, only about 10% of underweight and 9% of overweight adolescents had actively asked. While health care providers are suggested to always listen and seek to understand the views of patients with respect when addressing weight issues [45], little research has studied the effect of patients’ behaviour on doctor’s assessments. The positive effects of patient-doctor interactive relationship on weight assessment implied that adolescents especially those who are underweight /overweight should be educated to be more proactive about weight assessment by doctors.

Higher parental education level was associated with more assessments. Little literature has directly explored the association between parental education level and doctors’ assessments. Parents with higher education level were more likely to correctly perceive the weight status of overweight children compared with less educated parents [46]. However, it is unclear how parental education level affects the assessments by private practitioners. More studies are warranted to further address this gap.

Our study has several limitations. First, the assessments of weight-related factors were based on adolescent recall in the past 12 months, and the accuracy of these reports is unclear. A period of 12 months was set to include more adolescents; to facilitate their recall, each weight-related assessment item only required a simple binary response. Previous studies stated that data based on surveys of physicians could introduce selection bias of enrolling physicians who might be particularly interested in paediatric health, resulting in overestimation of the frequency of assessments [20,22,47]. Second, we assessed the frequency of assessments during the past 12 months, and the students could have seen several private practitioners, and thus the proportion estimated in the present study might be overestimated. Third, weight status might have changed since the medical consultation, which would attenuate the association between weight status and assessments. Fourth, recall bias was possible in that adolescents with greater weight concern had better recall of weight assessments. However, one study also reported that obesity was related to higher diet and higher exercise counseling rates using objective records of well-care child visit [21]. Fifth, due to the length of questionnaire, several potential confounders were not assessed. These included the presence of parents during medical consultation, length of medical consultation, and background characteristics of private practitioners [20,23-26]. Fifth, although the missing rate was as high as 36%, the subjects with missing data were generally comparable with those with missing data regarding sex, age, weight status, and highest parental education. The missing data probably did not bias the associations studied in the present paper, because the reasons of missing values in weight status and parental education is unlikely to be correlated with the assessments of weight-related factors, which were behaviours of private practitioners. In addition, results from the imputed data were generally similar to those from complete-case analysis. Last, since this is a cross-sectional study, cause-and-effect relation could not be assumed. We only have information on whether students received each assessment or not, and we recommend future research survey should also cover information on doctors’ assessments of obesity-related behaviours with frequency and intensity.

## Conclusions

Assessment of weight-related factors of adolescents by private practitioners was uncommon, and some adolescents actively asked private practitioners for advice on bodyweight. Adolescents who were female, underweight/overweight, had parents with higher education, and had actively asked a private practitioner for advice on weight were more likely to receive weight-related assessments. Since private practitioners may give weight status comments without objective measurements of BMI or WC, they will benefit from training or education regarding obesity screening.

## Competing interests

We have no financial relationships or other conflict of interest relevant to this article to disclose.

## Authors’ contributions

R H analysed the data and drafted the manuscript; SY H is the principle investigator of the HKSOS project and critically revised the manuscript; WS L coordinated the study and revised the manuscript; THL contributed to study design and supervision, and revised the manuscript. All authors read and approved the final manuscript.

## Pre-publication history

The pre-publication history for this paper can be accessed here:

http://www.biomedcentral.com/1471-2296/14/141/prepub
